# Dynamic biomarkers in hormone receptor-positive/HER2-negative breast cancer trials: a new hope for precision oncology

**DOI:** 10.1038/s41523-026-00904-5

**Published:** 2026-01-28

**Authors:** Giuseppe Di Grazia, Rodrigo Sánchez-Bayona, Climent Casals-Pascual, Tomás Pascual, Daniele Generali, Alessandra Gennari, Paolo Vigneri, Nadia Harbeck, Javier Cortés, Aleix Prat, Francesco Schettini

**Affiliations:** 1 Department of Human Pathology “G. Barresi”, University of Messina, Messina, Italy; 2 Translational Genomics and Targeted Therapies in Solid Tumors, August Pi i Sunyer Biomedical Research Institute (IDIBAPS), Barcelona, Spain; 3 Medical Oncology Department, Hospital Universitario 12 de Octubre, Madrid, Spain; 4 SOLTI Cancer Research Group, Barcelona, Spain; 5 Department of Clinical Microbiology, Biomedical Diagnostic Center (CDB), Hospital Clinic of Barcelona, Barcelona, Spain; 6 Barcelona Institute for Global Health (ISGlobal), Barcelona, Spain; 7 Faculty of Medicine and Health Sciences, University of Barcelona, Barcelona, Spain; 8 CIBER Enfermedades Infecciosas (CIBERINFEC), Instituto Salud Carlos III, Madrid, Spain; 9 Department of Medical Oncology, Clinic Barcelona Comprehensive Cancer Center, Barcelona, Spain; 10 SC Oncologia Multidisciplinare, ASST Cremona, Cremona, Italy; 11 Department of Medical, Surgery and Health Sciences, University of Trieste, Trieste, Italy; 12 Department of Translational Medicine, University of Piemonte Orientale, Novara, Italy; 13 Division of Medical Oncology, Maggiore University Hospital, Novara, Italy; 14 Department of Clinical and Experimental Medicine, University of Catania, Catania, Italy; 15 University Oncology Department, Humanitas Istituto Clinico Catanese, Misterbianco, Catania, Italy; 16 Breast Center, Department of Obstetrics and Gynecology and Comprehensive Cancer Center Munich, LMU University Hospital, Munich, Germany; 17 Oncology Department, International Breast Cancer Center (IBCC), Pangaea Oncology, Quironsalud Group, Barcelona, Spain; 18 Medica Scientia Innovation Research (MEDSIR), Barcelona, Spain; 19 Universidad Europea de Madrid, Faculty of Biomedical and Health Sciences, Department of Medicine, Madrid, Spain; 20 IOB Madrid, Institute of Oncology, Hospital Beata Maria Ana, Madrid, Spain; 21 Reveal Genomics, Barcelona, Spain

**Keywords:** Biomarkers, Cancer, Computational biology and bioinformatics, Oncology

## Abstract

Hormone receptor-positive/HER2-negative breast cancer evolves in response to therapy, demanding smarter, adaptive biomarker-based treatment strategies. We review emerging dynamic biomarkers to guide therapeutic decision-making, spanning tissue and liquid biopsies, metabolic imaging, and microbiome profiling, that capture tumor or host-related changes over time. By contrasting Academic and Industry approaches, we advocate for a cultural shift in clinical trial design and implementation, aiming to move from reactive to proactive Oncology.

## Introduction

**Fig. 1:**
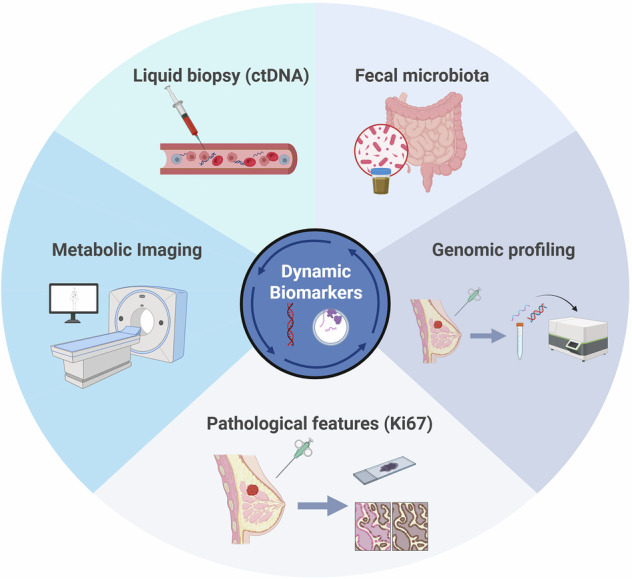
Overview of dynamic biomarkers in breast cancer. ctDNA circulating tumor DNA. Created in BioRender. Di Grazia, G. (2025) https://BioRender.com/snukyix.

Over recent decades, it has become clear that tumors are dynamic entities, evolving under therapeutic pressure through genomic, epigenomic, and host-related changes^1^. Advancing cancer treatment, therefore, requires biomarkers that can track these changes over time, monitoring response, anticipating resistance, and informing real-time clinical decisions^2,3^. This is no exception for hormone receptor-positive/HER2-negative (HR + /HER2-) breast cancer (BC), the most common breast malignancy. In this Perspective, we critically examine emerging dynamic biomarkers in HR + /HER2- BC (Fig. 1) and how academic and industry-led trials, through distinct yet complementary approaches, shape their development and integration into clinical practice.

## Tissue-based dynamic biomarkers: pathology vs. genomics

Clinical pathological features, such as Ki67 at diagnosis, have long guided treatment decisions and shaped patients’ prognosis in HR + /HER2- BC^4^. To note, intriguing evidence in recent years supports the use of Ki67 as a dynamic biomarker, with its changes serving as an indicator of sensitivity to endocrine therapy (ET)^5^. Among the first large-scale efforts to explore Ki67 dynamics in early BC (EBC), the randomized phase III trial POETIC elegantly investigated the short-term (2-week) use of perioperative aromatase inhibitors (POAI) to assess endocrine sensitivity *via* changes in Ki67^5^. The association with long-term outcomes was studied in nearly 4,500 postmenopausal women with HR + EBC^5^. Although no outcome difference was observed between patients receiving or not receiving POAI, the real innovation of the POETIC trial lay in the biomarker-driven design. By measuring Ki67 at baseline (Ki67_B_) and after 2-week POAI, the trial demonstrated that on-treatment Ki67 holds independent prognostic value, refining risk stratification beyond Ki67_B_^5^. For patients with HER2-disease in the POAI arm, 5-year recurrence risks dramatically increased from 4.3% in low-low Ki67 responders to 21.5% in those with persistently high Ki67, highlighting how a simple, time-bound pathological evaluation can serve as a dynamic indicator of endocrine sensitivity^5^. The ALTERNATE trial further reinforced the concept^6^. This phase III study enrolled postmenopausal women with HR + /HER2- EBC and compared neoadjuvant ET (NET) for up to 6 months, using on-treatment Ki67 levels to adapt therapy. The study introduced the Preoperative Endocrine Prognostic Index (PEPI) and on-treatment Ki67 as criteria to identify patients with endocrine-resistant disease early during NET. Patients with persistently high Ki67 (≥10%) at week 4 or 12 were classified as endocrine-resistant and discontinued ET, while those with PEPI = 0 after surgery were spared chemotherapy. Notably, among patients who completed NET, a substantial proportion achieved PEPI = 0, suggesting that dynamic endocrine sensitivity assessment may support chemotherapy omission in a biomarker-guided manner. These strategies innovatively underscored the clinical utility of dynamic tissue-based single-analyte markers in guiding adjuvant decisions. Nevertheless, implementation requires careful standardization and quality control.

The WSG-ADAPT trial extended evidence also to premenopausal patients and expanded on it by demonstrating that the combination of a baseline low-risk OncotypeDX result and dynamic endocrine response defined as Ki67 ≤ 10% after 3-4 weeks of preoperative ET allows omission of chemotherapy in N0-1 HR + /HER2- EBC, with excellent outcomes at a median follow-up of 60 months^7^. In the same line, the ADAPT-Cycle neoadjuvant trial enrolled patients with HR + /HER2- EBC at intermediate risk according to OncotypeDX and Ki67 response after 3-4 weeks of preoperative ET and randomized them to receive either two years of (neo)adjuvant ribociclib+ET or standard chemotherapy followed by ET^8^. Recently published preliminary results from the neoadjuvant-treated patients showed low but comparable pathologic complete response (pCR) rates between treatment arms, highlighting the limited benefit offered by chemotherapy in this population^9^.

Overall, these studies marked a conceptual shift, away from static prognostication and towards adaptive treatment strategies based on real-time tumor behavioral changes to individualize therapeutic decision-making.

Recently, more complex genomic biomarkers or profiles have significantly advanced the precision and temporal resolution of dynamic tumor characterization. The CORALLEEN trial exemplified this evolution, demonstrating how short-term CDK4/6-inhibitors+ET can elicit measurable transcriptomic shifts, captured through multigene signatures such as the PAM50-derived Prosigna Risk of Recurrence (ROR) score^10,11^. In this trial, postmenopausal women with stage I–IIIA HR + /HER2- BC with a luminal B intrinsic subtype (IS) by Prosigna^12^, were randomized to receive 6 months of neoadjuvant ribociclib+letrozole or standard anthracycline-taxane chemotherapy^10^. At baseline, 87% tumors were classified as ROR-high. However, after neoadjuvant treatment, nearly half transitioned to a ROR-low profile at surgery, reflecting the underlying proliferation suppression observed with both regimens^10^. ROR shifts were accompanied by broader genomic modulation, and IS changes in the same CORALLEEN and other studies^13–16^. Whether ROR itself may reflect such transcriptomic reprogramming is unclear. Nevertheless, ROR was used as a surrogate of molecular response, with the potential to support chemotherapy-sparing strategies in genomically-selected patients^10^. A similar philosophy underpins the design of the neoadjuvant RIBOLARIS trial (NCT05296746), which leverages transcriptional changes induced by 6-month ribociclib+letrozole to identify patients with an early transcriptional response at surgery using Prosigna ROR, potentially redefining the concept of in vivo endocrine sensitivity. In this trial, chemotherapy is omitted in molecular responders (ROR-low in surgery) or patients achieving pCR^17^.

Another compelling strategy is under investigation in patients with HR + /HER2- metastatic BC (MBC) treated with CDK4/6-inhibitor+ET, for whom gene expression and PAM50 IS are being assessed in biopsy samples obtained at tumor progression^18^. In a preliminary analysis, a shift towards less endocrine-sensitive, more proliferative subtypes, particularly HER2-enriched, was detected in 28% cases^18^. This subtype switch demonstrated prognostic significance regardless of posterior treatments. Furthermore, in patients whose tumors had evolved into non-luminal subtypes, chemotherapy-based regimens appeared to be more effective than endocrine strategies following progression^18^.

Finally, recent evidence from the POETIC trial revealed early transcriptomic reprogramming under NET, including intrinsic subtype changes, immune-modulation and downregulation of proliferation and estrogen receptor (ER)-related signatures^19^. These early transcriptional shifts correlated with survival outcomes better than baseline features^19^, suggesting that rapid transcriptomic changes could be tested to refine patient stratification and enable earlier therapeutic modulation during treatment. The examples provided highlight how dynamic genomic profiling, though more complex and resource-demanding than conventional pathological markers such as Ki67, offers a multidimensional perspective on tumor biology, reflecting broader pathway reprogramming and pharmacodynamic responses to treatment. How to translate this knowledge into therapeutic decisions remains to be elucidated in the context of ongoing and future clinical trials. Noteworthy, the use of standardized and reproducible genomic assays overcomes the limitation of the renowned inter-pathology variability associated with Ki67 and other pathology-based predictive biomarker assessments, such as HER2-low status and PD-L1^20,21^.

As genomic platforms continue to improve in standardization, reproducibility, cost-effectiveness, and clinical validation, their broader implementation should be actively pursued to advance Precision Oncology. Initiatives such as the SOLTI-HOPE study, which provides patients with access to the genomic profiling of their tumors in the metastatic scenario^22^, or the centralization of testing in reference laboratories, may help integrate these tools into routine therapeutic decision-making.

## Blood-based dynamic biomarkers: the liquid biopsy paradigm

Over the last decades, tissue-based approaches have represented the cornerstone of biomarker development in BC^23^. However, they are now being complemented, and in some contexts challenged, by liquid biopsy-based strategies. For instance, circulating tumor DNA (ctDNA) and circulating tumor cells (CTCs) offer the opportunity to capture tumor evolution in real time, overcoming the limitations of spatial and temporal heterogeneity intrinsic to single-site tissue biopsy, as well as the logistical and feasibility issues associated with it^24–26^, also enabling earlier detection of site-specific resistance that would likely be missed by conventional tissue biopsies^24,25,27^. Notably, ctDNA has emerged as the leading liquid biopsy tool due to higher sensitivity, broader coverage of inter-tumor heterogeneity, and suitability for earlier disease settings, whereas CTCs remain primarily prognostic and less informative in early-stage disease.

In the prospective randomized SUCCESS-A trial involving over 2,000 patients with EBC undergoing different adjuvant chemotherapy regimens, CTCs positivity before and after treatment was independently associated with worse prognosis^26^. In the IMpassion031 study testing neoadjuvant chemotherapy with or without atezolizumab, ctDNA clearance during treatment was associated with improved pCR and long-term outcomes^28^. Also in the early disease setting, ctDNA clearance after neoadjuvant therapy in HR + /HER2 − EBC was strongly associated with improved survival, suggesting that ctDNA dynamics might serve as an early surrogate of treatment efficacy and prognosis^29^. Moreover, several prospective studies demonstrated that ctDNA detection after surgery or completion of adjuvant therapy correlates strongly with increased risk of relapse, often preceding clinical-radiologic recurrence^30,31^. Overall, these findings support ctDNA monitoring as a real-time tool to assess treatment efficacy, stratify recurrence risk, and potentially trigger intervention in the molecular relapse window. However, results from ongoing trials such as SURVIVE and TREAT-ctDNA are awaited to prospectively evaluate whether early therapeutic interventions guided by blood-biomarker changes can truly improve patients’ outcomes^32,33^.

In the metastatic scenario, CTCs have emerged as a prognostic biomarker as well, correlating with progression-free survival (PFS) and overall survival (OS)^34^. Nonetheless, the SWOG S0500 trial prospectively demonstrated that CTCs' dynamics lack predictive value for guiding treatment changes^35^. Beyond enumeration, their molecular characterization may also offer insights into tumor evolution, resistance, and metastatic potential^36^. Still, no clear clinical utility for CTCs has been demonstrated so far. Concerning ctDNA, the academic phase III PADA-1 trial pioneered a molecularly guided strategy to anticipate endocrine resistance in HR + /HER2– MBC by longitudinally monitoring emerging *ESR1* mutations during first-line aromatase inhibitors (AI)+palbociclib^37^. *ESR1* mutations represent a well-characterized mechanism of acquired resistance to AI, leading to ligand-independent ER activation^38^. Among enrolled patients, ~27% developed ctDNA-detected *ESR1* mutations without radiologic progression; of these, 172 were randomized to early switch to fulvestrant+palbociclib versus continuing the same regimen. Median PFS nearly doubled with early switch (11.9 vs. 5.7 months), highlighting the potential of molecular progression as a therapeutic decision point^37^. Nevertheless, the absolute benefit over switching at radiologic progression was modest, and PADA-1 remains a conceptual step rather than a practice-changing approach.

More recently, the industry-led SERENA-6 phase III trial adopted a similar design, randomizing patients with ctDNA-detected *ESR1* mutations to continue AI or switch to the oral selective ER degrader (SERD) camizestrant while maintaining CDK4/6 inhibition^37,39^. Interim analyses favored camizestrant for PFS and patient-reported outcomes, supporting ctDNA-guided preemptive therapeutic adaptation^37,39^. However, the low proportion of patients ultimately randomized (9.7%), immature OS data, absence of crossover, costs of the seriated ctDNA detection strategy and limited drug availability currently restrict immediate clinical applicability. Together, these studies illustrate both the promise and the practical challenges of ctDNA-driven treatment strategies in HR + /HER2– MBC.

While the clinical potential of liquid biopsy is increasingly evident, the low sensitivity of current assays the technical variability of ctDNA/CTCs, and the lack of standardisation limit reproducibility and clinical reliability. Also, whether liquid biomarker–driven interventions can improve OS over radiologic-based standards remains unproven, with potential lead-time bias confounding their apparent benefit. Moreover, liquid biopsy does not inform on tumor microenvironment molecular and spatial features, lacks large-scale validation and is still frequently confined to selected cohorts or investigational use. Nonetheless, as costs decline and methodological harmonization and standardization advance, liquid biopsy is expected to play an increasingly complementary role alongside traditional tissue-based biomarkers.

## Imaging-based dynamic biomarkers: radiologic and metabolic response assessment

Radiologic imaging modalities have also been explored as dynamic biomarkers in HR + /HER2- EBC. Dynamic changes in tumor size measured by ultrasound or magnetic resonance imaging (MRI) during NET correlated with endocrine sensitivity, Ki67 changes, and long-term outcomes^40^. Additionally, BC metabolic activity might be monitored during treatment *via* imaging techniques to obtain early signals of therapeutic response, like elegantly achieved in the PHERGain phase II trial in HER2 + EBC^40^. Among ^18^F-FDG-PET responders treated with trastuzumab+pertuzumab alone, 38% achieved pCR, with significantly fewer adverse events and better quality-of-life compared to standard chemotherapy. A 3-year invasive disease-free survival rate of 94.8% in the PET-guided group further supported the feasibility and safety of a chemotherapy-free approach in a patients’ subset^41,42^. In HR + /HER2 − BC, a similar approach was tested in the single-arm PEARL phase 2 study, which preliminarily evaluated the efficacy of exemestane+everolimus using ^18^F-FDG-PET/CT as an early biomarker of response in a small cohort of 47 patients^43^. Molecular imaging was also combined with ctDNA dynamics analysis. The study found that patients with a metabolic response and/or undetectable ctDNA after 14 days of treatment had substantially better outcomes than non-responders. Although these findings require external validation, they highlight the potential of integrating functional imaging and molecular profiling to improve personalized treatment strategies in MBC. Alternatively, more specific imaging techniques for HR+ disease, such as ^18^F-fluoroestradiol(FES)-PET, have been developed, as well. FES-PET is a molecular imaging technique that visualizes ER expression in vivo, providing a non-invasive, whole-body assessment of ER activity and, similarly to liquid biomarkers, capturing heterogeneity across metastatic sites and enabling early detection of differential endocrine sensitivity^44^.

In HR + /HER2- BC, FES-PET could help identify patients most likely to benefit from endocrine-based therapies upfront, as successfully demonstrated in the EU-funded ET-FES academic trial^45^. Such molecular imaging could potentially guide de-escalation strategies by dynamically selecting those tumors with strong and homogeneous ER expression that may safely omit chemotherapy. As of now, it has been FDA-approved in 2020 and included in NCCN guidelines primarily for systemic staging in recurrent or metastatic HR+ disease, to assess ER expression in lesions that are difficult to biopsy or yield non-diagnostic results, and specifically for invasive lobular carcinoma due to its diffuse growth pattern and atypical metastatic behavior, supporting more accurate diagnosis and personalized treatment decisions^46^. A recent multidisciplinary consensus promoted by the European Association of Nuclear Medicine agreed on recommending FES-PET in HR + /HER2- BC, especially in cases of indeterminate findings after FDG-PET use^47^.

## Multidimensional biomarker integration

Another step towards improved Precision Oncology lies in the integration of multidimensional biomarkers to develop an adaptive, multilayered understanding of tumor evolution and treatment response. The I-SPY trial platform, particularly I-SPY2, illustrates the use of dynamic biomarkers in early BC. The study incorporates serial MRI, ctDNA monitoring, and molecular subtyping to assess treatment response in real time, enabling adaptive therapy decisions and providing a model for biomarker-driven precision oncology^48^. The SOLTI-PROMETEO I-II trials exemplify this approach by combining radiologic imaging, pathological assessment, and genomic profiling in the post-neoadjuvant scenario^49,50^. PROMETEO-I enrolled patients with HER2- BC with residual disease (RD) at MRI after standard NACT, biopsy-confirmed, who then received a short preoperative course of atezolizumab and Talimogene laherparepvec. This led to a 25% pCR rate and induced immune modulation (i.e., increased tumor-infiltrating lymphocytes, PD-L1-positivity and IGG gene signature), regardless of pCR^49^. PROMETEO-II focused on HR + /HER2- tumors with MRI-detected RD post-NACT, treating patients with 4 weeks of palbociclib+letrozole^50^. Complete cell cycle arrest (Ki67 ≤ 2.7%) was achieved in 59% of cases, alongside modulation of relevant immune and BC signatures^50^. These trials highlight the feasibility and clinical promise of integrating imaging, pathology, and molecular profiling to refine patient selection and enable real-time, biology-informed decisions. Still, the clinical actionability of these dynamic changes remains to be established.

## Fecal microbiota: a new frontier in dynamic biomarkers

Fecal microbiota (FM) has emerged as a promising, non-invasive dynamic biomarker with potential applications across various cancer types^51^. Increasing evidence suggests that gut microbiota composition may exert systemic pro-tumoral effects in HR + /HER2- BC and potentially interfere with therapeutic efficacy by modulating systemic inflammation, estrogen metabolism and cell survival and growth pathways through short-chain fatty acid and polyphenol metabolism^51^. Preclinical models have highlighted the immunomodulatory role of gut microbiota in shaping anti-tumor responses^51^ and antibody-dependent cellular cytotoxicity (ADCC)^51^. Preliminary studies in MBC have reported associations between specific bacterial species and treatment outcomes with chemo/immunotherapy^52^ or ET + CDK4/6-inhibitors^53^. Ongoing studies are exploring the potential of FM as a dynamic, host-derived biomarker, offering the key advantage of being non-invasive compared to tumor-based biomarkers.For instance, the BREAKFAST and BREAKFAST-2 trials, evaluating the impact of dietary interventions±metformin on the response to neoadjuvant therapy in triple-negative BC, also incorporated seriated FM assessments^54,55^. However, FM composition can be significantly influenced by host-related factors, including diet, proton pump inhibitor use, and antibiotic exposure, requiring careful study design and conduction^51,56^. Emerging evidence also indicates that intratumoral microbiota (IM) may play a significant role in cancer outcomes, complementing the FM role. Notably, BC has the highest bacterial load among cancers^57^. Its implications remain unclear, but further investigation could yield valuable insights.

## Academia vs. industry: different philosophies, complementary goals

Another key aspect in the development of dynamic biomarkers is the distinction between academic and industry-driven studies, particularly in their approaches and objectives. Academic trials often enjoy greater exploration, allowing researchers to delve into underlying molecular mechanisms using biological endpoints. However, their findings typically represent only an initial step towards clinical implementation, as these studies are frequently constrained by limited sample sizes and resources, which can compromise reproducibility and hinder standardization. Additionally, academic trials increasingly struggle with financial sustainability and timely execution, as they face burdensome bureaucracy, staff shortages, and chronic delays. Conversely, industry-sponsored studies are shaped by regulatory requirements and designed to ensure robustness, reproducibility, and clinical validation. While they often include companion diagnostics to support regulatory approval and clinical adoption, they tend to be cautious with biomarker-guided patient selection, as excessive stratification may restrict the therapeutic indication and limit the commercial potential of the drug. Moreover, the need to link a biomarker to a specific companion diagnostic can sometimes limit its widespread implementation, as it needs access to that exact platform, which may not be available in all centers, complicating clinical adoption and cross-trial comparability. Strictly regulatory-driven approaches may risk overlooking the underlying biological and clinical questions that truly need to be addressed, potentially leading to a proliferation of several biomarker-guided therapeutic options within the same disease setting without clear guidance on how to choose between them. The rising costs of novel therapies and technologies are rapidly becoming unsustainable for public health systems, leading to widening disparities in access to care and financial toxicity, this latter especially in systems where costs are largely covered by patients. In this context, the real challenge (and opportunity) lies in uniting academic and industry complementary efforts to promote the rational use of therapies and prioritizing clinically meaningful strategies. In this regard, a paradigmatic example can be represented by the I-SPY platform. I-SPY2 has advanced several investigational agents into industry-led phase 3 studies, illustrating how adaptive, biomarker-guided academic platforms can accelerate drug development. Its success, however, relies on complex Bayesian designs, substantial funding, industry collaboration, and organizational capacity, making it difficult to replicate widely. Nevertheless, public–private partnerships leverage the exploratory and mechanistic strengths of academia alongside the resources, regulatory expertise, and large-scale implementation capacity of industry.

## Validation, utility, approval, and recommendations

It is important to note that dynamic biomarkers are not at the same stage of validation. Their development requires sequential demonstration of analytical validity, clinical validity, and clinical utility, and only a few have progressed beyond the exploratory phase. Analytical validity has been demonstrated for most of them with regard to the methods used to detect them, regardless of the timing (e.g., Ki67 IHC assessment, Prosigna ROR score calculation, in-blood *ESR1* mutation detection with NGS, etc.). Regarding clinical validity, on-treatment Ki67 assessment for prognostication and potential treatment de/escalation has been tested in prospective trials. Other biomarkers, such as ROR downstaging or ctDNA-guided strategies, have been tested or are currently under investigation in prospective trials, as well. Others, such as fecal microbiome detection and its dynamic on-treatment assessment, are still in a very early stage of development, including the need for more analytical validation. Regarding clinical utility, guideline endorsement and regulatory approvals, the situation is quite heterogeneous. More details on analytical and clinical validity, clinical utility and regulatory approval and endorsement by major international guidelines of the discussed dynamic biomarkers are summarized in Table 1.

**Table 1:** Validation status, clinical setting, and regulatory approval of major dynamic biomarkers in HR + /HER2− breast cancer

Dynamic biomarker	Biomarker detection method	Analytical validity of the detection method	Clinical validity of the dynamic use of the biomarker	Clinical utility of the dynamic use of the biomarker	Regulatory/Guideline status for the dynamic use of the biomarker
On-treatment Ki67	IHC detection of Ki67 in FFPE tissues	Yes (but inter-operator variability)	Yes	Not uniformly accepted	Among the major international guidelines, only those mentioned by ESMO
Prosigna ROR changes	Gene expression assessment in FFPE tissue and score calculation	Yes	Under investigation	Under investigation	No
CTCs dynamics	CTC detection and isolation in plasma (different assays)	Assay-dependent (e.g., CellSearch yes, most others not)	Under investigation	Under investigation	No
ctDNA dynamics	Digital PCR, NGS-based platforms	Yes	Under investigation	Under investigation	No
On-treatment emergence of *ESR1* mutations in ctDNA	Digital PCR, NGS-based platforms	Yes	Yes	Under investigation	No
On-treatment functional imaging (FES-PET, FDG-PET)	PET	Yes	Under investigation	Under investigation	Approved for standard reassessment of the disease, but not to evaluate its dynamics for early treatment modulation
Fecal microbiome	Digital PCR, NGS-based platforms	Under investigation	Under investigation	Under investigation	No

*IHC* immunohistochemistry, *ESMO* European Society of Medical Oncology, *ROR* risk of recurrence, *CTCs* circulating tumor cells, *ctDNA* circulating tumor DNA, *FFPE* formalin-fixed paraffin-embedded, *FES-PET* ^18^F-fluoroestradiol positron emission tomography, *FDG-PET* ^18^F-fluorodeoxyglucose positron emission tomography, *PCR* polymerase chain reaction, *NGS* next-generation sequencing.

## Future directions and conclusions

**Fig. 2:**
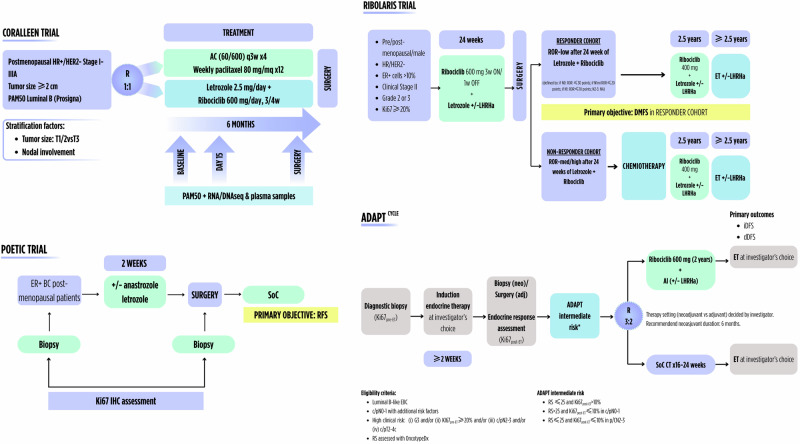
Examples of ongoing and completed clinical trials with tissue-based dynamic biomarkers. 3/4w 3 weeks out of 4, AC adriamycin + cyclophosphamide, AI aromatase inhibitor, BC breast cancer, CT chemotherapy, dDFS distant disease-free survival, DMFS distant metastases-free survival, EBC early breast cancer, ER+ estrogen receptor-positive, ET endocrine therapy, HR+/HER2- hormone receptors-positive/HER2-negative, IHC immunohistochemistry, iDFS invasive disease-free survival, LHRHa luteinizing hormone-releasing hormone analog, q3w every 3 weeks, R randomization, RFS relapse-free survival, ROR risk of recurrence, RS recurrence score, SoC standard of care. The figure has been generated with Canva (https://www.canva.com/es_es/pro/).

**Fig. 3:**
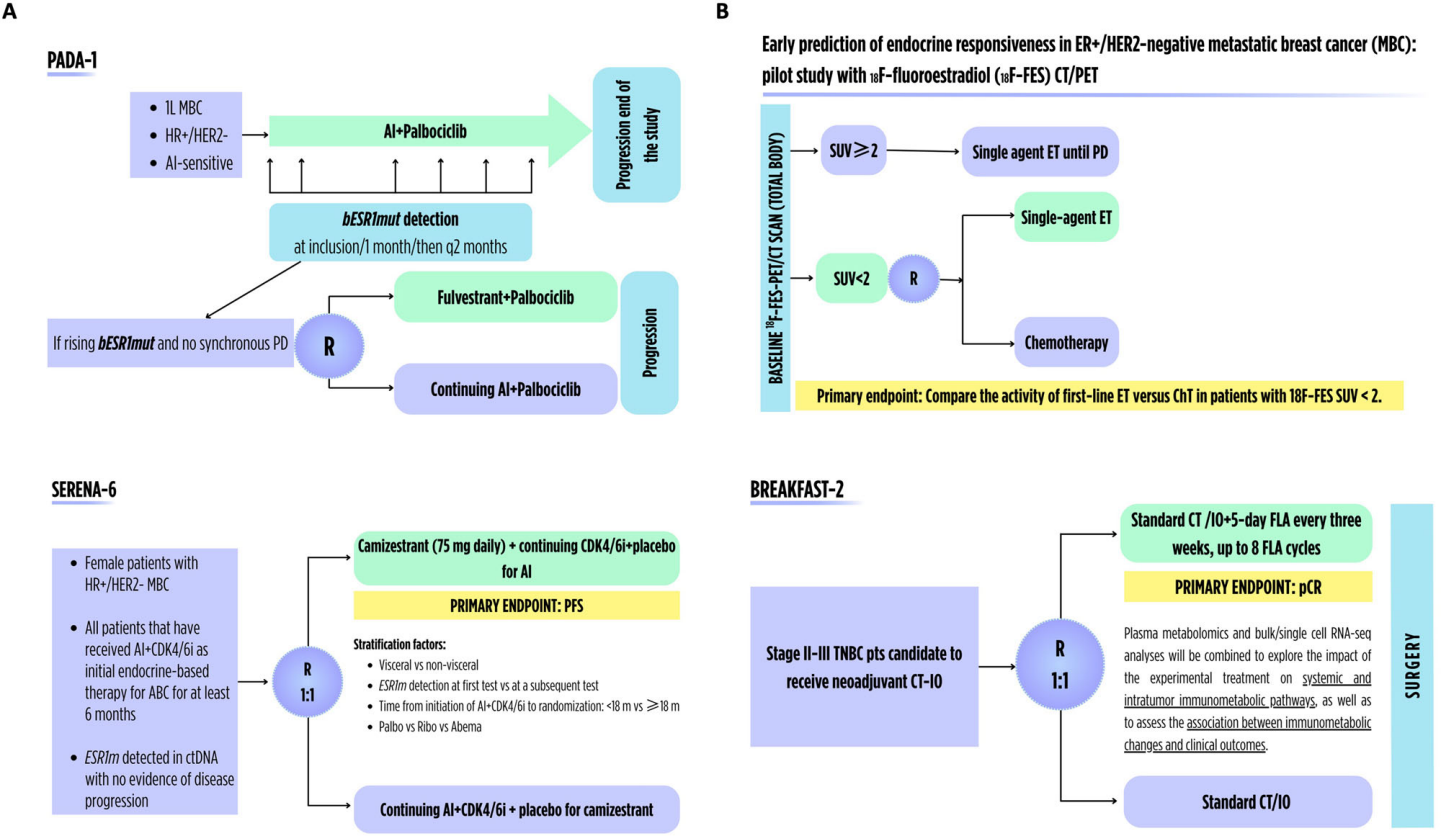
Examples of clinical trials focused on liquid biopsy-based therapeutic strategies and innovative metabolic imaging-based and fecal microbiota-based dynamic biomarkers. **A** Liquid biopsy-based trial; **B** Studies incorporating metabolic imaging and fecal microbiome. 1L first line, AI aromatase inhibitor, CDK4/6i cyclin-dependent kinase 4/6 inhibitors, CT chemotherapy, ER+ estrogen receptor positive, ET endocrine therapy, FLA fasting-like approaches, 18F-FES 16α-[18F]-fluoro-17β-estradiol, HR+/HER2- hormone receptors-positive/HER2-negative, IO immunotherapy, MBC metastatic breast cancer, pCR pathologic complete response, PD progressive disease, PET positron emission tomography, PFS progression-free survival, pts patients, q2 months: every 2 months, R randomization, SUV standard uptake volume, TNBC triple-negative breast cancer. The figure has been generated with Canva (https://www.canva.com/es_es/pro/).

The integration of dynamic biomarkers into the clinical management of HR + /HER2- BC represents an opportunity to evolve toward a more adaptive and biologically informed Oncology (Figs. 2 and 3)^58^. To unleash this potential, several strategic directions must be pursued. First, incorporating dynamic endpoints in clinical trials, either as exploratory or primary endpoints, will be essential. Traditional static measures, such as radiologic progression or pathologic responses, may not fully capture biological response or resistance dynamics. While baseline pathology biomarkers remain critical, they must be reinterpreted within a temporal and adaptive framework. However, it is important to recognize the limitations of longitudinal tissue-based biomarkers as well, including sampling bias due to spatial and temporal tumor heterogeneity and selection bias arising from the lack of usable tissue in patients with good responses. Integrating complementary approaches, when feasible, may help mitigate these biases and provide earlier, more sensitive indicators of therapeutic efficacy or failure.

Regulatory acceptance of such composite endpoints will require collaborative validation but could enable more efficient trial design.

Second, dynamic biomarkers must inform real-time therapeutic adaptation, as exemplified by the PADA-1 or SERENA-6^37,39^. This model of biomarker-driven intervention could expand to transcriptional classifiers, immune signatures, or resistance-associated CTC phenotypes, advancing from reactive to proactive Oncology. Importantly, technical, logistic, economic and clinical limitations should be properly addressed, so that biomarker-driven treatment changes are beneficial for patients and practical implementations simultaneously ensure economic sustainability, equitable access, clinical relevance and operational practicality.

Third, FM and IM are emerging as promising biomarkers. Yet, high interindividual variability, lack of methodological standardization, and potential confounding with host and environmental factors remain major challenges. Future strategies must implement robust standardization protocols, account for confounding variables and integrate microbiota profiling with other molecular and clinical data in multiparametric predictive frameworks.

Fourth, metabolic imaging may help monitor treatment response, but its applicability beyond trials is still uncertain, especially outside the HER2+ field. FES-PET holds promise for HR + /HER2- disease and should be further studied to define its role in monitoring therapeutic responses, especially to endocrine-based treatments.

Fifth, the development of clinical-biological decision algorithms will be key to implementing complexity at the bedside. These models should integrate tissue-based, circulating, microbial and imaging-based biomarkers with patient-specific clinical features and treatment histories to generate predictive and adaptive treatment recommendations. Continuous refinement through prospective data collection and real-world studies will be necessary to ensure generalizability and clinical utility. Additionally, efforts are ongoing to integrate multiple data layers, including imaging, pathology, molecular, and clinical features, using machine learning and artificial intelligence (AI) approaches. While promising for patient-specific predictions and therapy optimization, these strategies are still in early development^59,60^.

Finally, only through synergistic collaborations between academia and industry we can design trials that are both biologically informative and clinically impactful, accelerating the integration of dynamic biomarkers into standard-of-care.

To conclude, the future of Precision Oncology in HR + /HER2- BC requires a cultural shift in how we conceptualize, measure, and address tumor evolution in clinical trials and how we integrate validated dynamic biomarkers into every stage of patient care, ultimately moving from a static, baseline-driven classifications to an adaptive, multimodal, response-guided strategies that reflect the dynamic nature of BC under therapeutic pressure. While reactive in nature, by responding to early molecular signs of resistance or response, these biomarker-driven strategies embody a proactive philosophy that would enable the transition to a more personalized, adaptive, biology-driven Oncology.

## Data Availability

No datasets were generated or analyzed for the present article.
